# Advances in Detection Techniques for the H5N1 Avian Influenza Virus

**DOI:** 10.3390/ijms242417157

**Published:** 2023-12-05

**Authors:** Xianshu Fu, Qian Wang, Biao Ma, Biao Zhang, Kai Sun, Xiaoping Yu, Zihong Ye, Mingzhou Zhang

**Affiliations:** Zhejiang Provincial Key Laboratory of Biometrology and Inspection & Quarantine, College of Life Science, China Jiliang University, 258 Xueyuan Street, Xiasha Higher Education Zone, Hangzhou 310018, China; fxs@cjlu.edu.cn (X.F.); 19550160980@163.com (Q.W.); mb@cjlu.edu.cn (B.M.); zb@cjlu.edu.cn (B.Z.); sunkai@cjlu.edu.cn (K.S.); yxp@cjlu.edu.cn (X.Y.); zhye@cjlu.edu.cn (Z.Y.)

**Keywords:** H5N1 subtype, avian influenza virus, detection technology

## Abstract

Avian influenza is caused by avian influenza virus infection; the H5N1 avian influenza virus is a highly pathogenic subtype, affecting poultry and human health. Since the discovery of the highly pathogenic subtype of the H5N1 avian influenza virus, it has caused enormous losses to the poultry farming industry. It was recently found that the H5N1 avian influenza virus tends to spread among mammals. Therefore, early rapid detection methods are highly significant for effectively preventing the spread of H5N1. This paper discusses the detection technologies used in the detection of the H5N1 avian influenza virus, including serological detection technology, immunological detection technology, molecular biology detection technology, genetic detection technology, and biosensors. Comparisons of these detection technologies were analyzed, aiming to provide some recommendations for the detection of the H5N1 avian influenza virus.

## 1. Introduction

Avian influenza (AI) is an infectious disease caused by avian influenza virus (AIV) infection; it exhibits various symptoms, ranging from effects on the respiratory system to severe systemic sepsis. According to two surface glycoproteins of AIV, the antigens of hemagglutinin (HA) and neuraminidase (NA) can be divided into different subtypes; so far, 16 HA subtypes (H1–H16) and 9 NA subtypes (N1–N9) have been identified [1]. AIV can infect and reproduce in macrophages and lymphocytes, leading to the destruction of lymphocytes [2] and causing a series of respiratory and gastrointestinal symptoms. In mid-February 2009, a swine flu outbreak (H1N1 influenza virus) broke out in Mexico, causing a widespread epidemic, but the symptoms of most infected individuals were mild [3]. H5N1 AIV can be transmitted from birds to humans, and the mortality rate is extremely high [4]. Since 2003, 17 countries around the world have reported 861 cases of human infection with H5N1 avian influenza to the World Health Organization (WHO). In addition, most cases have occurred in Asian countries, and the mortality rate of all cases exceeds 50% [5].

In 1996, H5N1 AIV was initially isolated from geese in Guangdong province [6], and human cases of H5N1 AIV infection were reported in 1997 in a poultry outbreak in Hong Kong, China. Since then, H5N1 AIV has continued to spread among poultry populations and has had a serious impact on the global poultry farming industry [7]. Since 2003, H5N1 AIV has dominated poultry in southern China and Southeast Asia [8]. With the global contagion of H5N1 AIV, which has had a growing impact on wild birds [9], in the summer of 2021, an outbreak of H5N1 avian influenza in several populations in Scotland resulted in the deaths of many breeding Great Skuas (*Stercorarius skua*) [10]. The seasonal migration of *Stercorarius skua* may lead to its spread to other areas. In December 2021, poultry deaths occurred on a farm in St. John’s, Canada, due to infection with H5N1 avian influenza [11]. In January 2022, on the west coast of Namibia, many double-crested cormorants (*Phalacrocorax auritus*) died on account of infection with H5N1 avian influenza [12]. On January 9, 2023, the World Health Organization received a report that Ecuadorians were infected with H5N1 avian influenza [13]. The years 2014–2015 and 2022–2023 represented two major disasters for poultry. The dominant subtype is H5N1, so it is highly important to study the detection technology used to detect H5N1 AIV.

To date, scientists have developed many detection methods for the detection of H5N1 AIV; however, there is still space for improvement in the operability, sensitivity, and on-site applicability of the existing detection methods. This paper compares and analyzes serology, immunology, molecular biology, gene detection techniques, and biosensors to develop a detection technology that is more accurate, more convenient, and more easily applied on-site compared to existing methods for the detection of H5N1 AIV.

## 2. Virus Isolation and Identification

Virus isolation and identification are the “gold standard” for the detection of AIV [14]. Virus isolation and identification can recognize the representative characteristics of all epidemic subtypes of AIV [15]. The traditional method involves culturing the AIV from specific pathogen-free (SPF) eggs or cells and then detecting it via virus isolation [16]. The respiratory secretions, brain tissue, blood, and spleens of diseased chickens can be selected as pathological materials, sterilized with saline or a buffer solution in a certain proportion, and put into a grinder for grinding; a suspension is made. After centrifugation, antibiotics are added to the supernatant to filter out the miscellaneous bacteria; then, the processed pathological material is inoculated through the allantoic cavity into 9–11-day-old SPF chicken embryos; it is incubated at 37 °C, and the chicken embryo allantoic fluid is collected. Then, the agar diffusion test or hemagglutination (HA) and hemagglutination inhibition (HI) test are selected for virus identification. Although virus isolation and identification constitute the “gold standard” for detecting AIV, they require relatively lengthy detection times. Shan et al. [17] found that, in the most diluted concentration, the virus culture method requires three days to confirm the presence of the virus in the sample, and it requires fresh and refrigerated samples to obtain optimal sensitivity. In addition, the samples must be stored at 2–8 °C and processed within 2–3 days to prevent the excessive loss of the virus titer. Although virus isolation and identification are obviously more sensitive than antigen detection methods [18], the isolation of H5N1 AIV needs to be performed in a Biosafety Level 3 Laboratory (BSL3), and laboratory safety measures must be strengthened, such as the use of respiratory equipment; additionally, all laboratory waste must be removed, and personnel must shower before leaving, etc. These measures are in place to prevent possible biosafety problems. Since the number of BSL3 laboratories is relatively small [19,20], this detection method is not suitable for rapid and large-scale detection.

## 3. Serological Detection Technology

### 3.1. HA and HI Test

The HA and HI test is an economical, rapid, and widely used detection method [21]. This method is used to check whether the allantoic fluid collected after virus isolation exhibits hemagglutination activity; then, antiviral serum is used to perform hemagglutination inhibition tests on the isolate in need of hemagglutination activity identification [22]. Wiriyarat et al. [23] proved that red blood cells from guinea pigs and geese represent the best hemagglutination assay for the AIV. The HA and HI test is highly sensitive and specific and is considered the gold standard for the detection of antibodies to H5N1 AIV in serodiagnosis [24]. It can be used to identify specific antibodies of different subtypes, but cross-reactions may occur between subtypes [25,26]. When detecting H5N1 AIV antibodies in human serum, it exhibits low sensitivity [27].

### 3.2. Enzyme-Linked Immunosorbent Assays (ELISA)

To date, many enzyme-linked immunosorbent assays have been developed to detect antibodies to the AIV in poultry, as well as other species including humans [28]. Most of them comprise indirect ELISA detection, requiring specific anti-immunoglobulin conjugates. ELISA also requires concentrated and purified live virus preparations to be used as test antigens [29,30,31]. A. L. Shafer et al. [32] developed a competitive ELISA (CELISA) based on the recombinant baculovirus of AIV for the serological diagnosis of AIV in various birds. This CELISA does not require the use of several anti-immunoglobulin conjugates or live viruses, avoiding the potential risk of infection from exposure to live viruses. ELISA can detect cross-reactions between different subtypes of AIV [33,34]. For the detection of H 5N1 AIV, ELISA has higher specificity and sensitivity compared with the detection of the microneutralization method [27]. Zhang et al. [35] developed a double-antibody sandwich ELISA (DAS-ELISA) for AIV nucleoprotein (NP). This method uses monoclonal antibodies (MAb) as capture antibodies and horseradish peroxidase-labeled rabbit-derived polyclonal immunoglobulin G (IgG) as detection antibodies; they can detect H1-H15 subtypes AIV and exhibit no cross-reaction with other avian pathogens. Chen et al. [36] established a simple and rapid instrument for the ELISA detection of H5N1 AIV. Compared with virus isolation and identification, the overall prediction rate of this method is 96.6%, and it is suitable for field detection. Ho et al. [37] established an antigen capture ELISA (AC-ELISA) based on H5 hemagglutinin-specific monoclonal-antibody-targeting conformational epitopes and N1 neuraminidase linear epitopes, which can concurrently detect H5 and N1 subtype antigens; it is used for the rapid detection of H5N1 AIV, and this method could be applied to the double-site detection of clinical or environmental samples. Prabakaran et al. [38] established an epitope-blocking ELISA (EB-ELISA), which can be used to detect specific antibodies to H5N1 AIV in human or animal serum. This detection method relies on a monoclonal antibody that can bind to the epitope of H5 hemagglutinin. The EB-ELISA can easily detect H5N1 antibodies in the sera of immunized animals or convalescent humans and has 100% specificity. Compared to HA and HI and trace neutralization, this method exhibits higher sensitivity and specificity. He et al. [39] developed a dot ELISA (H5-Dot ELISA) detection method for the detection of the H5 subtype AIV, using two complementary monoclonal antibodies, Mab 6B8, and Mab 4C2, to identify arginine and lysine at position 189 of H5N1 AIV, as well as asparagine and serine at position 155. The hemagglutinin protein of H5N1 AIV contains a specific polybasic cleavage site rich in arginine; Dong et al. [40] established a detection method for H5N1 AIV at this site. Overall, ELISA has the advantages of high sensitivity and specificity, but the ELISA detection method has poor reproducibility [41].

## 4. Immunological Detection Technology

### 4.1. Collagen Gold Immunochromatography Technology

**Figure 1:**
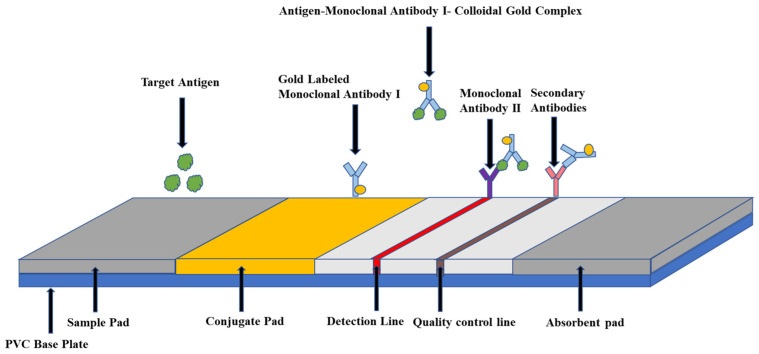
Principles of collagen gold immunochromatography technology.

Colloidal gold immunochromatography is a lateral flow chromatography technique that uses colloidal gold as the label. The principle of Colloidal gold immunochromatography is shown in Figure 1. The colloidal gold-labeled antibody is adsorbed on the binding pad. When the tested antigen is added to the sample pad at one end of the test strip, it moves forward via capillary action, and they react with each other after dissolving the colloidal gold label on the binding pad. The antigen-antibody complex formed by the binding of the gold-labeled antibody to the detection line combines with the monoclonal antibody and gathers on the detection line, and the color development results can be observed by the naked eye [42]. Li et al. [43] established a colloidal gold immunochromatographic technique for the rapid semi-quantitative detection of H5 AIV. In this method, four test lines with different concentrations were designed to reflect the degree of virus infection in different organs. In general, in the clinical field, especially in developing countries, the strip based on a lateral flow chromatography strip constitutes an alternative and cheap diagnostic tool [44]. However, the examiner can only complete simple technical operations and lacks the scientific information necessary to rationally evaluate the clinical value and reliability of the test results and the detection method [45].

### 4.2. Fluorescence Immunodetection Technique

In fluorescence immunochromatography, the coated antibody or antigen is used as the detection line, the secondary antibody is used as the quality control line, the fluorescent-labeled antibody or antigen is fixed on the connecting pad, and the tested sample is moved on the chromatographic strip via capillary action. The markers used in fluorescence immunoassays include fluorescent quantum and fluorescent nanomaterials, which can replace conventional enzyme markers and colloidal gold as labeling materials. Durairaj et al. [46] developed a rapid detection method for the H5N1 virus based on cysteamine-gold coated carboxylated fluorescent europium nanoparticles, which can be used for field inspections. Moreover, the potential surface charge and fluorescence stability of such fluorescent nanomaterials can improve the detection limit by about eightfold. Zhang et al. [47] used the sandwich hybridization of quantum dots and magnetic beads to establish a new method for the detection of H5N1 AIV, in which quantum dots were coupled with oligonucleotide probes and fluorescent signals were generated, while magnetic beads (MBs) were coupled with probes to separate and concentrate signals. This assay requires only 0.1 ng of viral RNA to detect avian influenza subtypes. Yeo et al. [48] developed a rapid dual-fluorescence diagnostic system (SRDFDS) based on quantum dot bioconjugate (QD) components and smartphones. The sensitivity of the fluorescent system for H5N1 AIV detection was 78.57% (11/14), and the specificity was 97.37% (37/38). Using two pairs of HA protein MAb tests marked with fluorescent quantum dots as an antibody, Clsa et al. [49] established the H5 bird flu virus fluorescence immune chromatography test. This method can detect the H5N1 avian flu virus with 100 times more specificity than commercial H5 bird flu virus colloidal gold test strips. In general, traditional fluorescent dyes have poor stability and insufficient fluorescence intensity. In comparison, quantum dots have unique optical advantages such as high fluorescence intensity, a wide excitation spectrum, adjustable fluorescence emission size and composition, a high quantum yield, and less photobleaching [50]. However, at present, quantum dot labeling has high equipment requirements and costs.

## 5. Molecular Biological Detection Technology

### 5.1. Reverse-Transcription Polymerase Chain Reaction (RT-PCR)

**Figure 2:**
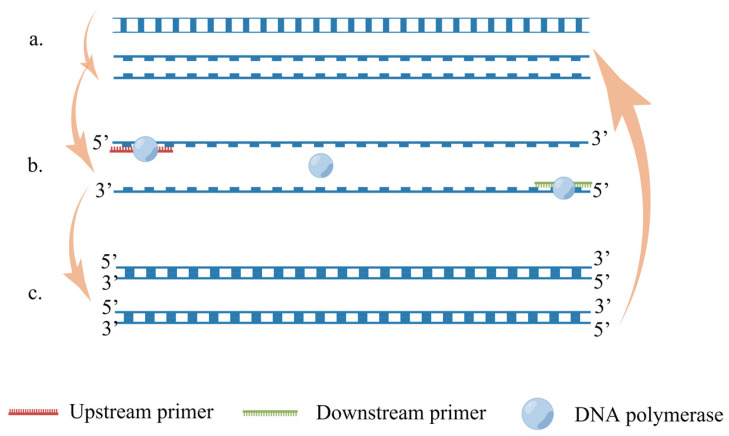
Principles of PCR. (By Figdraw, https://www.figdraw.com): (**a**) DNA template thermal deformation: DNA double-stranded unraveling; (**b**) complementary sequence pairing and binding between the primer and a single strand of template DNA; (**c**) synthesis of new strands under the action of DNA polymerase.

A polymerase chain reaction (PCR) is a method for the in vitro amplification of specific DNA fragments; it is widely used in microbial detection, veterinary medicine, aquaculture, and many other fields. PCR technology is based on the known DNA sequence, using two oligonucleotides as primers, in an in vitro enzymatic amplification reaction [51]. The amplification principle of PCR is shown in Figure 2. RT-PCR is the synthesis of cDNA from mRNA under the catalysis of reverse transcriptase, which is subsequently used as a template for the amplification reaction using specific primers [52]. Payungporn et al. [53] invented a one-step multiplex RT-PCR method, which designed three groups of primers for the M gene, H5 gene, and N1 gene for multiple amplification. This method is carried out in a single tube in one step, which significantly reduces the risk of nucleic acid contamination, takes less time, and has good specificity. There was no cross-reaction with other subtypes. Chen et al. [54] established a real-time quantitative RT-PCR method, which designed universal primers according to the HA gene, and this method was more accurate than routine RT-PCR. Real-time quantitative RT-PCR has higher sensitivity compared with ELISA; it is more efficient and less time-consuming than virus isolation and identification detection methods. However, the sensitivity of the real-time quantitative RT-PCR method is affected by factors such as RT-PCR inhibitors in samples, inefficient RNA extraction procedures, and the rapid degradation of RNA before detection. Park et al. [55] designed three sets of primers and probes for the H5, N1, and N8 genes and established a multiplex real-time RT-PCR method for the simultaneous detection of H5N1 and H5N8 subtypes of AIV. This method was as sensitive as a single RT-PCR method, but 10 times less sensitive than the SYBR Green real-time RT-PCR method [56]. At present, real-time fluorescent RT-PCR is the preferred method for the detection of mRNA [57]. Real-time fluorescent RT-PCR is an RT-PCR system that includes SYBR Green, TaqMan probes, etc. The real-time fluorescent RT-PCR has higher sensitivity and specificity than RT-PCR and can be quantified [58]. Kis et al. [59] established a real-time RT-PCR method combined with TaqMan probes, which effectively identified four different branches of H5N1 AIV originating from Vietnam, but this method is not the standard method for detecting H5N1 AIV. Ellis et al. [60] developed and validated a one-step real-time RT-PCR method based on TaqMan probes for the detection of H5N1 AIV, and it can obtain specific results within a few hours. Lu et al. [61] established real-time RT-PCR combined with TaqMan Minor Groove Binder (TaqMan-MGB) probes. This method uses direct fluorescence detection instead of gel electrophoresis, which can shorten the detection time, and TaqMan-MGB probes are more stable than conventional TaqMan probes. SYBR Green I is simpler and cheaper than fluorescent probes because it can be used with any set of amplification primers [62]. Naguib et al. [63] developed the SYBR Green real-time fluorescent RT-PCR detection method for H5N1 AIV; it showed results that were fully consistent with the sequence analysis when using RNA from virus isolates and was also suitable for the rapid screening of clinical samples. RT-PCR plays an important role in infectious disease surveillance due to its good specificity, high sensitivity, and rapid results [64], and it has been extensively applied in the detection of H5N1 AIV. However, it is highly prone to producing false positives or negatives if the technical operations or laboratory environment are substandard.

### 5.2. Recombinase Polymerase Amplification (RPA)

**Figure 3:**
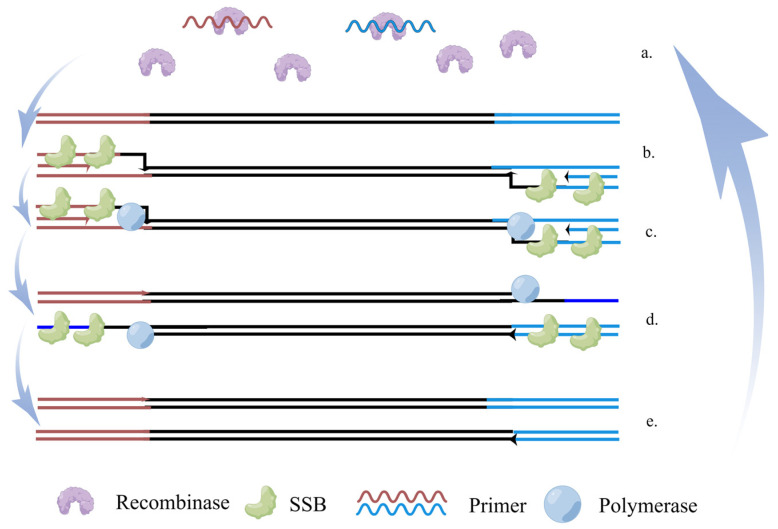
The principle of RPA (By Figdraw.): (**a**) primer binding to recombinase; (**b**) strand displacement; (**c**) polymerase-guided new-chain synthesis; (**d**) homoduplex separation; (**e**) two duplexes form.

RPA was developed by Piepenburg et al. [65] in 2006. Its basic principle is similar to that of PCR, except that the thermal cycle in PCR is combined with the single strand by the recombinase in RPA [66]. The amplification principle of RPA as illustrated in Figure 3. Yehia et al. [67] designed primers and probes for RPA based on the HA gene of H5N1 AIV and established a detection method for H5 RT-RPA with 100% sensitivity, which can run in five to seven minutes. The time it takes to detect an RNA molecule is much shorter than the 60–90 min of real-time RT-PCR. Wang et al. [68] combined EXO probes to establish the RT-RAA detection method, which can detect various H5 subtypes of AIV, including H5N1 subtypes. The RPA detection method does not require special instruments. Compared with other DNA amplification technologies, it can react at a lower temperature. The combination of RPA and test strips is more suitable for on-site detection. In addition, RPA also has the advantages of convenient operations and reactions. It has the advantages of rapidity, the easy visualization of results, and low requirements for instruments and technicians [69]. Compared with other isothermal techniques, RPA is considered to be an ideal platform for POCT because of its high efficiency, low temperatures, and easy operation. However, primer design in RPA analysis remains a significant bottleneck, because, apart from some guiding methods, there are no specific description rules, automatic software design cannot be carried out [70], and the best primers need to be screened through experiments.

### 5.3. Loop-Mediated Isothermal Amplification (LAMP)

**Figure 4:**
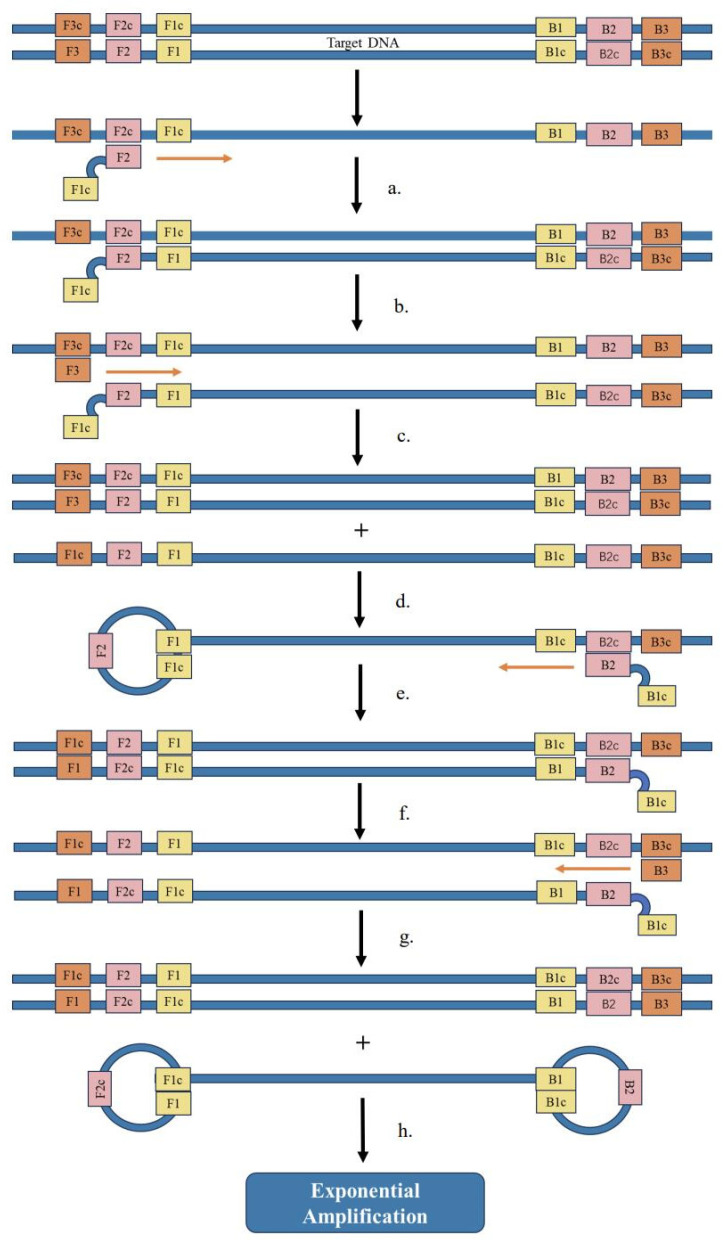
The principle of LAMP: (**a**) the F2 sequence of the primer combines with the F2c region of the template to synthesize complementary chains with the template; (**b**) the F3 sequence of the primer is combined with the F3c region of the template to extend and initiate chain substitution; (**c**) formation of new double chains and replacement of single chains; (**d**) the replaced single-chain F1 region complements the F1c region to form a circular structure, the replaced single-stranded F1 region complements the F1c region to form a circular structure, and the primer B2 sequence complements and extends the single-stranded B2c region; (**e**) formation of a new double chain; (**f**) the primer B3 sequence complements, extends, and initiates chain substitution with the B3c region of the template chain; (**g**) formation of new double chains and replaced single chains, with complementary F1 sequences and F1c sequences at both ends of the replaced single chain, and complementary B1 sequences and B1c sequences, forming a circular structure; (**h**) exponential amplification. The orange and black arrow indicate the direction of amplification and the amplification process, respectively.

Notomi et al. [71] developed a novel isothermal nucleic acid amplification approach in 2000, namely, LAMP. This method carries out the amplification reaction under the isothermal condition of about 65 °C, and it only takes about 30 min to complete all the reaction steps. It does not require a template thermal denaturation process or a long-term temperature cycle, and this method does not require any expensive materials. With advanced instruments and reagents, the reaction can be completed using only a water bath [72]. In addition, this method produces a large amount of white magnesium pyrophosphate precipitates during the reaction [73], which can be detected by a turbidimeter, so it is suitable for on-site detection. The amplification principle of LAMP as shown in Figure 4, where the orange and black arrow indicate the direction of amplification and the amplification process, respectively. Imai et al. [74] used reverse-transcription loop-mediated isothermal amplification (RT-LAMP), a distinctive nucleic acid testing technique; they designed two internal primers and two external primers and established a set of fast and sensitive tests. Compared with ordinary RT-PCR, the method used to detect H5N1 AIV, the sensitivity of this system is 100 times higher. Jayawardena et al. [75] established an RT-LAMP method for the detection of H5 AIV from viral cultures without RNA extraction; however, the limit was approximately 1000-fold lower than that for the detection of purified RNA. Dinh et al. [76] developed an improved RT-LAMP for the detection of H5N1 AIV and designed a set of primers with higher specificity and sensitivity than previously reported. Jung et al. [77] established a method combining multiple RT-LAMP with immunochromatographic test strips and designed two outer primers and two inner primers targeting the HA gene. Two loop primers and five primers were designed according to the M gene, which can detect not only H5N1 AIV but also H1, H3, H5, and other subtypes at the same time; thus, it is suitable for the early detection of AIV on site. Tang et al. [78] established an immunoassay-based RT-LAMP (Immuno-RT-LAMP) assay for the rapid detection of H5N1 AIV in whole-blood samples; H5N1 AIV was specifically captured from blood samples, and, after a thermal lysis, they were exponentially amplified on a real-time PCR instrument or in a water bath system for quantitative analysis. Zhang et al. [79] designed primers based on the M gene, the H5 gene, and the H9 gene of AIV and established RT-LAMP technology, which can screen out AIV and distinguish the H5 and H9 subtypes; the detection limit is 10 times that of RT-PCR, and the detection time is less than 30 min. RT-LAMP detection is a promising diagnostic tool that can be used for disease detection, does not require complex, expensive equipment or trained personnel [80], and is highly suitable for on-site detection. However, this detection method has targeting-related disadvantages; it is more difficult to design specific primers for the target sequence.

### 5.4. Nuclear Acid Sequence-Based Amplification (NASBA)

**Figure 5:**
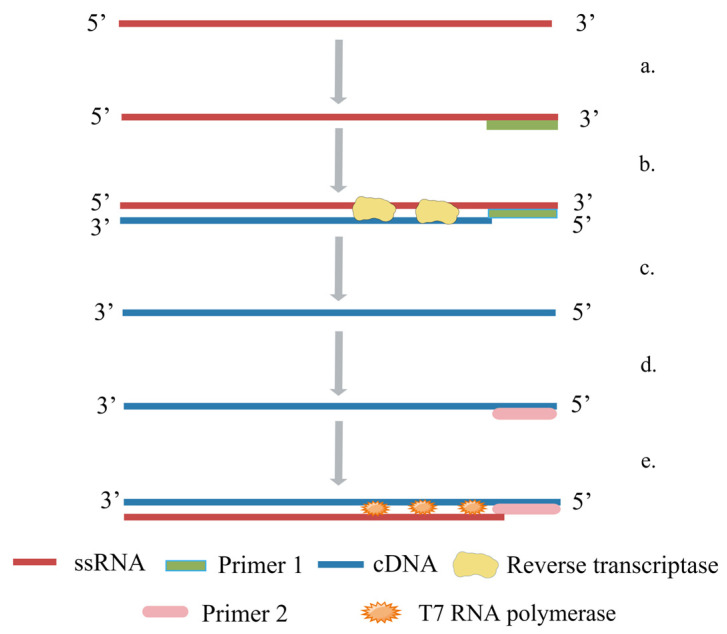
The principle of NASBA (By Figdraw.): (**a**) Primer 1 binds to the 3′ end of the template chain; (**b**) synthetic cDNA; (**c**) RNase H breaks down RNA template chains; (**d**) Primer 2 binds to the 5′ end of DNA; (**e**) T7 RNA polymerase synthesizes RNA chains.

NASBA is an enzyme-based single-step RNA amplification reaction [81]. The reaction necessitates the presence of three enzymes: avian myelocytic disease virus reverse transcriptase (AMV-RT), ribonuclease-H, and T7 RNA polymerase. The design of two target-sequence-specific primers is necessary, one of which should have a 5′ extension containing the T7 promoter sequence of the RNA polymerase. The amplification primarily involves single-stranded RNA and a primer-free RNA synthesis step [82]. The amplification principle of NASBA is illustrated in Figure 5. NASBA technology offers several advantages, including the direct recombination of the RNA target without the need for reverse transcription into cDNA. Furthermore, it enables the selective amplification of RNA sequences in the presence of natural genomic DNA [83]. Collins et al. [84] established a NASBA detection method for the Eurasian H5 subtype of AIV; it can be used not only for detecting H5N1 subtype AIV but also for distinguishing highly pathogenic AIV from lowly pathogenic ones. Moore et al. [85] developed a real-time NASBA detection method targeting the H5 and N1 genes; the average turnaround time from sample receipt to reporting results was four hours, and its sensitivity is comparable to that of RT-PCR. Shan et al. [86] employed NASBA for the detection of H5 AIV vaccines and observed that this method exhibited comparable specificity and sensitivity to virus isolation and identification. Shan et al. [17] compared NASBA with virus isolation and found that NASBA is more suitable for detecting anal swabs. Chantratita et al. [87] found that the positive detection rate of NASBA was higher than that of RT-PCR. In the detection of H5N1 AIV, NASBA performed very well in terms of specificity, the positive detection rate, and sensitivity. This technology has also been used in the detection of new coronaviruses and Zika viruses [88]; because of its simple operation and good specificity, it has broad application prospects. However, it is currently less commonly used in the detection of AIV.

## 6. Gene Testing Technology

### 6.1. Gene Chip

In gene chip technology, many synthetic DNA probes are fixed on the surface of the chip carrier in an orderly manner to produce a gene chip. After the sample to be tested is amplified and labeled, it hybridizes with the gene chip, and the gene information of the sample under examination can be obtained by scanning and analyzing the signal of the hybridization reaction [89]. Kessler et al. [90] fixed oligonucleotide probes in silicon microchannels, including the H1, H3, and H5 hemagglutinin genes and the N1 and N2 neuraminidase genes; this not only detected AIV but also distinguished subtypes. Dawson et al. [91] developed a single-gene diagnostic chip that showed 95% sensitivity and 92% specificity in a clinical trial of 53 H5N1 AIV. Lee et al. [92] developed a low-density gene chip to detect H5, H7, H9, N1, N2, and N3 and evaluated the diagnostic chip using six high-pathogenicity H5N1 clade 2.3.2 AIV from outbreaks in South Korea during the winter of 2010–2011. It was found that this method had great potential for the rapid typing of high-pathogenicity AIV. In the study conducted by Shi et al. [93], a gene chip is established; the method uses the RT-PCR amplification of the H5 H7, and H9 genes and the N1, N2, and M genes, marking it on the aldehyde slides on DNA chips. This method can simultaneously detect many kinds of AIV, including the H5N1 subtype. Kwon et al. [94] A quantitative reverse-transcription polymerase chain reaction (RT-qPCR) diagnostic method based on gene chips has been developed for the rapid identification of multiple AIVs, including H5 subtypes. In general, gene chip technology reduces the occurrence probability of non-specific reactions caused by increasing multiple primers to detect multiple pathogens at the same time in traditional detection technology. This technique exhibits significant advantages in the diagnosis of mixed infection with multiple pathogens [95], but this detection technology has high costs.

### 6.2. Next-Generation Sequencing (NGS)

NGS is a new sequencing method comparable with first-generation DNA sequencing technology [96]. In 1977, Sanger et al. [97] invented the first-generation sequencing technology, and it has been widely applied, but its sequencing reaction time is long, the sequencing throughput is low, and the cost is high; it is not suitable for the detection of multiple samples [98]. Pyrophosphate sequencing is a high-throughput non-gel DNA sequencing method introduced at the end of 1990s. It adopts a real-time sequencing method based on the pyrophosphate synthesis DNA released by incorporation of dNTP [99]. Ellis et al. [60] established a detection method combining TaqMan RT-PCR and pyrosequencing analysis, which sequenced the amplicon of RT-PCR to detect H5N1 AIV. In general, high-throughput sequencing technology has the advantages of high throughput and high sensitivity and may be the most accurate method. It is widely used in the detection of various pathogens, but it is more time-consuming and difficult than RT-PCR and requires expensive equipment. Therefore, gene sequencing is mainly used for a research tool rather than a primary diagnostic method [100].

## 7. Biosensor

### 7.1. Surface Plasmon Resonance (SPR)

**Figure 6:**
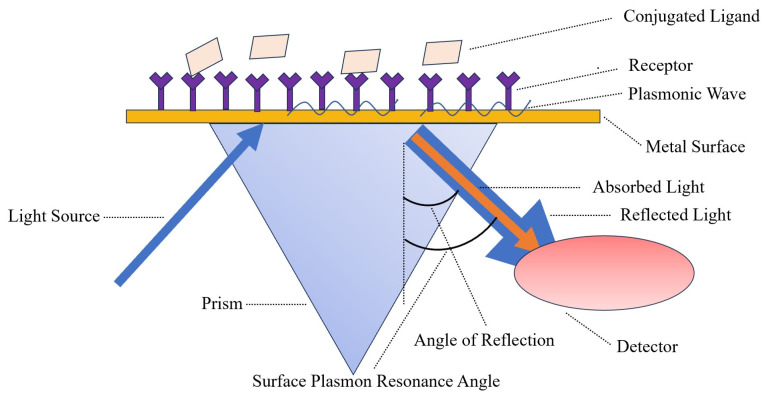
The light source of the SPR biosensor is polarized light, and the sensing chip is coated with a layer of gold film. In the experiment, a target molecule is fixed on the surface of the gold film, and then the molecules that interact with it are dissolved in the solution and flow through the chip surface, causing the SPR angle to change accordingly.

Surface plasma technology was introduced to the sensor boundary in the early 1980s; it can be described as a collective oscillator in free-electron plasma at the metal boundary [101]. SPR biosensors are mainly composed of three components: an optical waveguide device, a metal film, and a biomolecular film [102]. The detection principle of SPR is identified in Figure 6. Bai et al. [103] used biotinylated DNA aptamers as specific recognition elements for portable SPR biosensors, and then fixed them on the gold surface of the sensor coated with streptavidin through a combination of streptavidin and biotin. Fixed aptamers capture the H5N1 avian influenza virus, increasing the refractive index (RI). The sensor was able to detect H5N1 AIV with a concentration of 0.128–12.8 HAU within 1.5 h. Wong et al. [104] established an SPR biosensor for the detection of H5N1 AIV antibody biomarkers, which requires neither time-consuming interference fringe analysis nor any extraction process; it is unlabeled and works in real-time. In general, SPR biosensors have the advantages of being label-free, highly sensitive, and high throughput. However, because SPR is based on the measurement of reflected light, if the tested sample is Turbidite, the detection result will be affected.

### 7.2. Field-Effect Transistor (FET)

FET is a semiconductor device that utilizes electric field effects to control output current [105], with high input resistance (107–1015 Ω), low noise, large dynamic range, low power consumption, wide safe working area, and easy integration [106]. Gao et al. [107] established a novel semiconductor silicon nanowire FET (SiNW FET) biosensor that can detect viral nucleic acid in real-time; it can detect DNA as low as 1fM. Moreover, it has high specificity for single-base errors and can selectively detect H1N1 and H5N1 AIV at the same time. Guo et al. [108] fabricated indium tin oxide thin film transistors on glass substrates. Through 3-glycidyl oxygen propyl, silane will increase the oxygen radicals of the H5N1 avian flu virus monoclonal antibodies, which are fixed on the ITO channel; the introduction of the H5N1 virus affects the ITO TFT’s electronic properties, which generates the threshold voltage and leads to the change in the field-effect mobility. Kwon et al. [109] established a labeled FET-free biosensor for the detection of AIV in chicken serum, in which DNA aptamers are used as receptors for hemagglutinin proteins immobilized on gold microelectrodes. The specific binding of target proteins leads to changes in surface potential, resulting in a signal response of field-effect transistor transducers. In the range of 10 pM to 10 nM, it increases linearly with the logarithmic concentration of HA protein, with a detection limit of 5.9 pM. Sensors based on field-effect transistors have shown high selectivity and stability in H5N1 AIV sensing and are rapidly developing towards more sensitive, miniaturized, simpler, and more portable models [110]. However, the sensitivity and accuracy of field-effect biosensors still need to be improved, and the requirements for operators are relatively high.

### 7.3. Electrochemical Biosensor

Electrochemical biosensors are sensors based on the electrochemical characteristics of the object being measured; It converts the chemical quantity of the object to be tested into electrical quantities for sensing and detection. It is composed of a biological recognition body and an electrochemical transducing system, in which the biological recognition body has the function of specifically identifying various tested substances, and the transducing system can convert biochemical signals into electrical signals [111,112]. Kukol et al. [113] investigated a label-free detection system based on electrochemical impedance spectroscopy; it is capable of detecting the DNA sequence of an H5N1 AIV hybridized to a single-stranded DNA oligonucleotide probe linked to a gold surface. This method is simpler to perform than traditional EIS and is also able to analyze environmental samples for the presence of AIV. Liu et al. [114] developed an electrochemical biosensor for detecting the genetic sequence of H5N1 AIV with DNA aptamer immobilized on electrodes modified with mixed nanomaterials, using multi-walled carbon nanotubes (MWNT), polypyrrole (PPy) nanowires (PPNWs), and gold nanoparticles (GNPs) to assemble and modify the electrode to improve its selectivity and sensitivity. Shi et al. [115] successfully constructed a label-free electrochemical biosensor for the detection of H5N1 AIV and prepared a highly directed hybrid microarray on a gold substrate with good specificity and stability. Jarocka et al. [116] built a device based on a glassy carbon electrode electrochemical immunosensor; using a protein, they achieved resistance to its monoclonal antibody, recombinant His6 H5 HA antigen, and BSA-modified glassy carbon electrodes. Not only can it be used for the determination of the existence of the H5 monoclonal antibody in the blood in the buffer, but it also can be used to detect serum antibodies against H5 HA chicken. Diba et al. [117] established a sandwich detection platform containing surface-formed aptamer–protein–antibody complexes by introducing a new DNA aptamer/H5N1 protein/ALP-labeled anti-H5N1 sandwich complex on a gold-nanoparticle-modified carbon electrode. The amperometric detection of H5N1 AIV proteins can be achieved with high selectivity and sensitivity. Fang et al. [118] developed an unlabeled electrochemical sensor, which uses the H5N1 avian influenza virus gene sequence as the target DNA and [Fe (CN) _6_]^3−/4−^ solution as an electrochemical indicator. DNA aptamers were self-assembled on flower-shaped VS2, graphene, and Au nanoparticle-modified glassy carbon electrodes. Has good stability and repeatability. Fu et al. [119] developed an electrochemical resistance biosensor based on carbon nanotubes to detect H5N1 AIV. Using nitrogen-doped multi-walled carbon nanotubes (N-MWCNTs) and semiconducting single-walled carbon nanotubes (sc-SWCNTs) as active sensing elements, this biosensor is small, flexible, and easy to use. In general, the electrochemical biosensor has the advantages of strong specificity, good stability, and repeatability, but there are testing problems such as high costs and short lifespans.

### 7.4. Gene Biosensor

The principle of a gene biosensor is to hybridize the probe fixed on the surface of the sensor or transducer probe with another complementary ssDNA molecule; the double-stranded DNA will show a certain physical signal, which will finally be reflected by the transducer [120]. Lee et al. [121] developed a label-free biosensor for DNA hybridization detection that was not affected by small changes in the reference voltage; they successfully achieved the specific detection of the oligonucleotide sequence of H5N1 AIV. Grabowska et al. [122] developed a single-electrode genetic sensor capable of the simultaneous determination of the HA and NA sequences of H5N1 AIV, using two different oligonucleotide probes covalently fixed to a gold electrode surface through Au-S bonds. One probe was complementary to the cDNA of H5 with ferrocene modification at its 5′ end (SH-ssDNA-Fc), and the second probe was decorated with methylene blue at its 5′ end (Complementary cDNA of SH ssDNA MB and N1). This dual-gene sensor was selective and had similar detection limits for both genes. Grabowska et al. [123] established an electrochemical gene sensor with a reduction–oxidation (REDOX)-labeled oligonucleotide probe of metal carboborane [3-iron diterpene] on a gold electrode. The 5′ end of the probe was modified with an NH2 group and covalently attached to the electrode with 3-mercaptopropanoic acid SAM. The system is highly sensitive to targets containing sequences complementary to the probe and reacts very weakly to non-complementary targets. Malecka et al. [124] developed a genetic sensor for detecting specific DNA and RNA sequences of H5N1 AIV, achieving good selectivity. Malecka et al. [125] studied a gene based on a gold electrode electrochemical sensor, which exists in the sample solution of REDOX active markers [Fe (CN)_6_]^3−/4−^. Using a square-wave voltammetry analysis of signals, the gene sensor has good sensitivity and selectivity; what is more, it is easy to make. In general, genetic biosensors have good specificity and accuracy, but, at the same time, such biosensors are not suitable for field detection due to their complex design and significant environmental influence.

### 7.5. Impedance Biosensor

**Figure 7:**
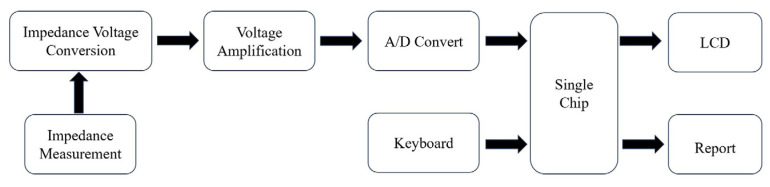
Principles of impedance biosensors.

The impedance biosensor is placed into the corresponding detection medium, and a specific-frequency and small-amplitude AC potential wave is used to measure the ratio of the AC potential to the current signal, which is the impedance of the system and will change with the change in the measurement frequency [126]. The detection principle of impedance biosensors is displayed in Figure 7. Yan et al. [127] established an impedance biosensor based on interdigitated array microelectrodes (IDAMs) combined with immunomagnetic separation. Fix magnetic nanospheres coated with streptavidin onto biotin-labeled anti-H5 monoclonal antibodies, capture H5 AIV from the sample solution through specific immune reactions, and then separate and concentrate it using a magnetic field. From sampling to detection completion, impedance measurement of interdigital array microelectrodes in the frequency range of 20 Hz to 1 MHz can be completed in only 1.5 h. Lin et al. [128] designed and manufactured a separator based on a high-intensity, high-gradient magnetic field to improve previous portable impedance biosensor instruments, making them faster and more reliable. This improved impedance biosensor can recognize the H5N1 avian influenza virus within 30 min. Lum et al. [129] designed an improved impedance biosensor in which polyclonal antibodies of the N1 subtype are immobilized on the microelectrode surface and specifically bind to the H5N1 avian influenza virus. Chicken red blood cells are used as biomarkers for capturing the H5N1 avian influenza virus to amplify impedance signals. The improved impedance biosensors have higher specificity and reliability and shorter testing times. Lin et al. [130] developed a wet etching technology using the improved impedance of the sensor; the cost is lower than the dry etching process, and a new compact electrode design is used to increase microelectrode production and reduce the cost of the impedance biosensors. Lum et al. [131] selected DNA aptamers by phylogenetic evolution of index enriched ligands (SELEX) and immobilization of biotinylated aptamers targeting H5N1 AIV onto the electrode surface through biotin–streptavidin binding. The H5N1 avian influenza virus is captured on the surface of the microelectrode, causing an increase in impedance, and detection can be completed within 30 min.

## 8. Conclusions

Early diagnosis is crucial to controlling the spread of H5N1 AIV in good time. The methods used for detecting H5N1 AIV have been developing in the direction of more convenient, faster, more accurate, and more sensitive techniques. The comparison of sensitivity, specificity, and detection limit of various detection techniques is exhibited in Table 1. Virus isolation and identification is the most accurate method, but it is time-consuming and involves biosafety risks. It can only be carried out in laboratories of tertiary grade and above and cannot be used for field detection. Hemagglutination and hemagglutination inhibition tests are difficult to carry out and cannot be used to detect large quantities of samples. ELISA is simple to operate, but its sensitivity and specificity are lower than virus isolation and identification, and the repeatability is not good. Although the colloidal gold immunoassay is convenient, the results cannot be quantified, the accuracy is insufficient, and the test results are sometimes unreliable. Fluorescence immunochromatography is more sensitive than colloidal gold strips, but the fluorescence signal can easily be interfered with, and the accuracy of quantification cannot be guaranteed. Detection methods involving molecular biology have good specificity and sensitivity, but they have high requirements in terms of experimental equipment and operators. In recent years, more and more biosensors have been used for the detection of H5N1 AIV, and significant progress has been made. Many new materials have been used as biosensors, which reduces the cost of manufacturing of biosensors, but there is still room for improvement in sensitivity and capture probes. In the future, the detection technology used for the H5N1 subtype of AIV will continue to develop in the direction of more convenient, faster, more accurate, higher-sensitivity, and lower-cost methods, and the interdisciplinary application of multi-disciplinary technology represents another future development trend in detection technology.

## Figures and Tables

**Table 1:** Comparison of sensitivity, specificity, and detection limit of various detection techniques.

Category	Technology	Sensitivity (%)	Specificity (%)	LOD	Reference
Serology	ELISA	95	100	2.5 × 10^2^ EID50	[36]
Immunology	(1) Collagen Gold Immunochromatography Technology	100	100	1 mL of allantoic fluid (HA titer 1:27) continuously diluted 1:100–1:1000	[132]
	(2) Fluorescence Immunodetection Technique	78.57	97.37	80 HAU/mL	[49]
Molecular biology	(1) RT-PCR	80	100	100 copies/reaction	[56]
	(2) RPA	97.26	100	102cRNA copies/μL	[69]
	(3) LAMP	100	87.5	1 PFU/tube	[75]
	(4) NASBA	-	-	10 copies/reaction	[89]
Genetics	Gene chip	97	100	-	[93]
Biosensor	(1) SPR	-	-	0.128–12.8 HAU	[105]
	(2) FET	-	-	1fM	[109]
	(3) Electrochemical biosensor	-	-	5.2 × 10^−14^ M	[120]
	(4) Gene biosensor	-	-	100pM	[123]
	(5) Impedance biosensor	-	-	0.25 HAU	[133]
